# Mitochondrial cytochrome P450 1B1 is involved in pregnenolone synthesis in human brain cells

**DOI:** 10.1016/j.jbc.2023.105035

**Published:** 2023-07-11

**Authors:** Yiqi Christina Lin, Garett Cheung, Zeyu Zhang, Vassilios Papadopoulos

**Affiliations:** Department of Pharmacology and Pharmaceutical Sciences, Alfred E. Mann School of Pharmacy and Pharmaceutical Sciences, University of Southern California, Los Angeles, California, USA

**Keywords:** steroidogenesis, human glial cells, pregnenolone, CYP1B1, neurosteroid, cytochrome P450

## Abstract

Neurosteroids, which are steroids synthesized by the nervous system, can exert neuromodulatory and neuroprotective effects *via* genomic and nongenomic pathways. The neurosteroid and major steroid precursor pregnenolone has therapeutical potential in various diseases, such as psychiatric and pain disorders, and may play important roles in myelination, neuroinflammation, neurotransmission, and neuroplasticity. Although pregnenolone is synthesized by CYP11A1 in peripheral steroidogenic organs, our recent study showed that pregnenolone must be synthesized by another mitochondrial cytochrome P450 (CYP450) enzyme other than CYP11A1 in human glial cells. Therefore, we sought to identify the CYP450 responsible for pregnenolone production in the human brain. Upon screening for CYP450s expressed in the human brain that have mitochondrial localization, we identified three enzyme candidates: CYP27A1, CYP1A1, and CYP1B1. We found that inhibition of CYP27A1 through inhibitors and siRNA knockdown did not negatively affect pregnenolone synthesis in human glial cells. Meanwhile, treatment of human glial cells with CYP1A1/CYP1B1 inhibitors significantly reduced pregnenolone production in the presence of 22(R)-hydroxycholesterol. We performed siRNA knockdown of CYP1A1 or CYP1B1 in human glial cells and found that only CYP1B1 knockdown significantly decreased pregnenolone production. Furthermore, overexpression of mitochondria-targeted CYP1B1 significantly increased pregnenolone production under basal conditions and in the presence of hydroxycholesterols and low-density lipoprotein. Inhibition of CYP1A1 and/or CYP1B1 *via* inhibitors or siRNA knockdown did not significantly reduce pregnenolone synthesis in human adrenal cortical cells, implying that CYP1B1 is not a major pregnenolone-producing enzyme in the periphery. These data suggest that mitochondrial CYP1B1 is involved in pregnenolone synthesis in human glial cells.

Steroids have important functions in the central nervous systems (CNS) and peripheral nervous systems, including modulation of behavior, pain, stress, and inflammation. Although it was initially thought that steroids were synthesized in the peripheral steroidogenic tissues and then transported into the central nervous system, it has now been accepted that the brain can produce steroids *de novo* as well (1). These locally synthesized steroids, known as neurosteroids, regulate physiological processes such as neural development, reduction of neuroinflammation, and neuroprotection even in the absence of peripherally derived steroids (2, 3, 4). Progestogens, estrogens, and androgens have all been shown to dampen proinflammatory cytokines to modulate neuroinflammation, which are implicated in traumatic brain injury and neurodegenerative diseases such as Alzheimer’s disease (5). Neurosteroids can alter gene expression *via* genomic actions as well as allosterically modulate neurotransmitter receptors *via* nongenomic actions (6). For example, allopregnanolone is a potent positive allosteric modulator of GABA_A_ receptors that has been shown to have anxiolytic effects, allowing it to be translated into the first approved neurosteroid drug, indicated for postpartum depression (7).

Classical steroidogenesis begins with conversion of cholesterol to pregnenolone by the cytochrome P450 11A1 (CYP11A1) enzyme in the mitochondria (8). This enzymatic reaction occurs in three steps: two hydroxylation steps to make the intermediates 22(R)-hydroxycholesterol and 20α,22(R)-dihydroxycholesterol, followed by cleavage of the side chain by the desmolase activity of CYP11A1. Pregnenolone is not only the precursor required to make all other neurosteroids but is also an active neurosteroid itself with important functions in memory, neuroplasticity, anti-inflammation, and neuroprotection (see (9) for review). For example, pregnenolone can promote neuron tubulin assembly and myelination, which are important in recovery after nerve injury (10, 11). Pregnenolone has also been shown to enhance memory in multiple rodent models, as well as alleviate learning deficits induced by β-amyloid, ethanol, and tetrahydrocannabinol (12, 13, 14, 15, 16). It should be noted that pregnenolone is more potent than its metabolites (pregnenolone sulfate, dehydroepiandrosterone (DHEA), DHEA sulfate) at inducing these procognitive effects (12, 13), indicating its importance as a neurosteroid rather than merely a precursor to its steroid metabolites. In clinical trials, pregnenolone has shown therapeutic potential in schizophrenia, bipolar depression, and chronic pain disorders (17, 18, 19). Given that pregnenolone is an active neurosteroid and the major precursor of neurosteroids, understanding its biosynthetic pathway is essential. Indeed, alterations in pregnenolone will not only directly influence physiological processes but also alter the levels of downstream neurosteroids and the brain functions that they modulate.

Even though pregnenolone is the most abundant steroid in the human brain (20), there are discrepancies in the literature regarding its biosynthetic pathway. The enzyme that synthesizes pregnenolone, CYP11A1, has been difficult to detect in rodent brains and especially in human brains (see (21) for review). In our recent study, we found extremely low levels of *CYP11A1* mRNA in human brain tissues and cells, with undetectable levels of CYP11A1 protein (22). However, human glial cells synthesized detectable levels of pregnenolone despite undetectable CYP11A1 protein, and pregnenolone production can be enhanced through the addition of the CYP11A1 substrate 22(R)-hydroxycholesterol (22(R)-HC) but not inhibited by CYP11A1 inhibitors. We also found that iron chelation and knockdown of the mitochondrial cytochrome P450 (CYP450) co-factor ferredoxin reductase (FDXR) significantly reduced pregnenolone synthesis in the glial cells, suggesting that another mitochondrial CYP450 other than CYP11A1 is responsible for pregnenolone production in the human brain.

CYP450s are oxygenases that play important roles in metabolizing xenobiotics, sterols, fatty acids, vitamins, and other molecules (23). These enzymes contain a heme group that accepts electrons from NADPH to be used in catalytic reactions and are generally considered membrane-bound, with majority of CYP450s found in the endoplasmic reticulum (ER). However, seven CYP450s are found only in the mitochondria. Mitochondrial CYP450s require cofactors ferredoxin and FDXR for their activity, while ER-localized CYP450s use the co-factor NADPH-CYP450 reductase (POR) instead (24). Out of the seven mitochondrial CYP450s, we proposed that CYP24A1 and CYP27 enzymes are likely candidates for the enzyme responsible for pregnenolone synthesis in the brain (22). Apart from the classical mitochondrial, CYP450s, CYP1A1, CYP1B1, CYP2B1/2, CYP2D6, CYP2D7, CYP2E1, and CYP2U1 have been reported to have some mitochondrial localization in addition to localizing to the ER (25, 26). While the classical mitochondrial CYP450s have an N-terminus mitochondria-targeting sequence, the CYP450s that localize to both the ER and mitochondria have a bimodal targeting signal at the N-terminus (26). This bimodal targeting signal consists of an ER-targeting sequence followed by a mitochondria-targeting sequence, with a protease cleavage site in between the two targeting sequences. Depending on the physiological demand of the cell, cytosolic proteases or protein kinases can cleave the bimodal targeting sequence to expose the mitochondria-targeting signal and preferentially localize the enzyme to the mitochondria.

Building on our previous findings (22), the goal of the current study is to identify the specific mitochondrial CYP450 used by human glial cells for synthesis of pregnenolone. We screened for CYP450s that could be involved in pregnenolone production and identified CYP27A1, CYP1A1, and CYP1B1 as potential enzymes to investigate further.

## Results

### Screening of CYP450s in human glial cells

We generated a list of CYP450 genes that are expressed in the human brain based on publicly available microarray data (27) and designed primers for each of these genes (Table S1 and Dataset S1). Since CYP11A1 levels in the human brain are too low to be responsible for the levels of pregnenolone found in the brain (21, 22), we aimed to identify CYP450s that have higher expression than CYP11A1. Using quantitative reverse transcription PCR (qRT-PCR), we evaluated the expression of various CYP450s in Miyazaki glioblastoma multiforme-1 (MGM-1), Miyazaki glioblastoma multiforme-3 (MGM-3), normal human astrocyte (NHA), and human microglia clone 3 (HMC3) cells (Table 1, Dataset S1), which are different human glial cell lines that we have previously shown to synthesize pregnenolone independently of CYP11A1 (22). MGM-1 and MGM-3 cells have oligodendrocyte phenotypes, NHA cells are astrocytes, and HMC3 cells are microglia. We used MGM-1 cells as our main model for the current study, as oligodendrocytes produce more pregnenolone than other glia (28, 29). We found that five CYP450 genes satisfy the criteria of having mitochondria localization and higher expression than *CYP11A1* in the human glial cells: *CYP27A1, CYP1A1, CYP1B1, CYP2U1,* and *CYP2E1* (Table 1). We chose to exclude *CYP2U1* and *CYP2E1* from further investigations because their relative RNA expressions are not notably higher than that of *CYP11A1* in the glial cell lines. Furthermore, CYP2U1 and CYP2E1 are known to be involved in the metabolism of fatty acids (30, 31, 32) rather than cholesterol or steroids like CYP27A1, CYP1A1, and CYP1B1.

**Table 1:** Relative RNA expression of cytochrome P450s with mitochondrial localization and higher expression than *CYP11A1* in human glial cells

MGM-1		MGM-3		NHA		HMC3	
Gene	Expression	Gene	Expression	Gene	Expression	Gene	Expression

*CYP27A1*	0.00150	*CYP1B1*	0.03561	*CYP1B1*	0.01266	*CYP2U1*	0.00015
*CYP1B1*	0.00125	*CYP27A1*	0.00133	*CYP2U1*	0.00095	*CYP1B1*	0.00011
*CYP2U1*	0.00049	*CYP2U1*	0.00040	*CYP2E1*	0.00039	*CYP2E1*	0.00009
*CYP1A1*	0.00007	*CYP1A1*	0.00024	*CYP27A1*	0.00014	*CYP1A1*	0.00005
*CYP2E1*	0.00005	*CYP11A1*	0.00003	*CYP11A1*	0.00005	*CYP11A1*	0.00001
*CYP11A1*	0.00001						

### CYP27A1 does not appear to be responsible for pregnenolone synthesis

**Figure 1:**
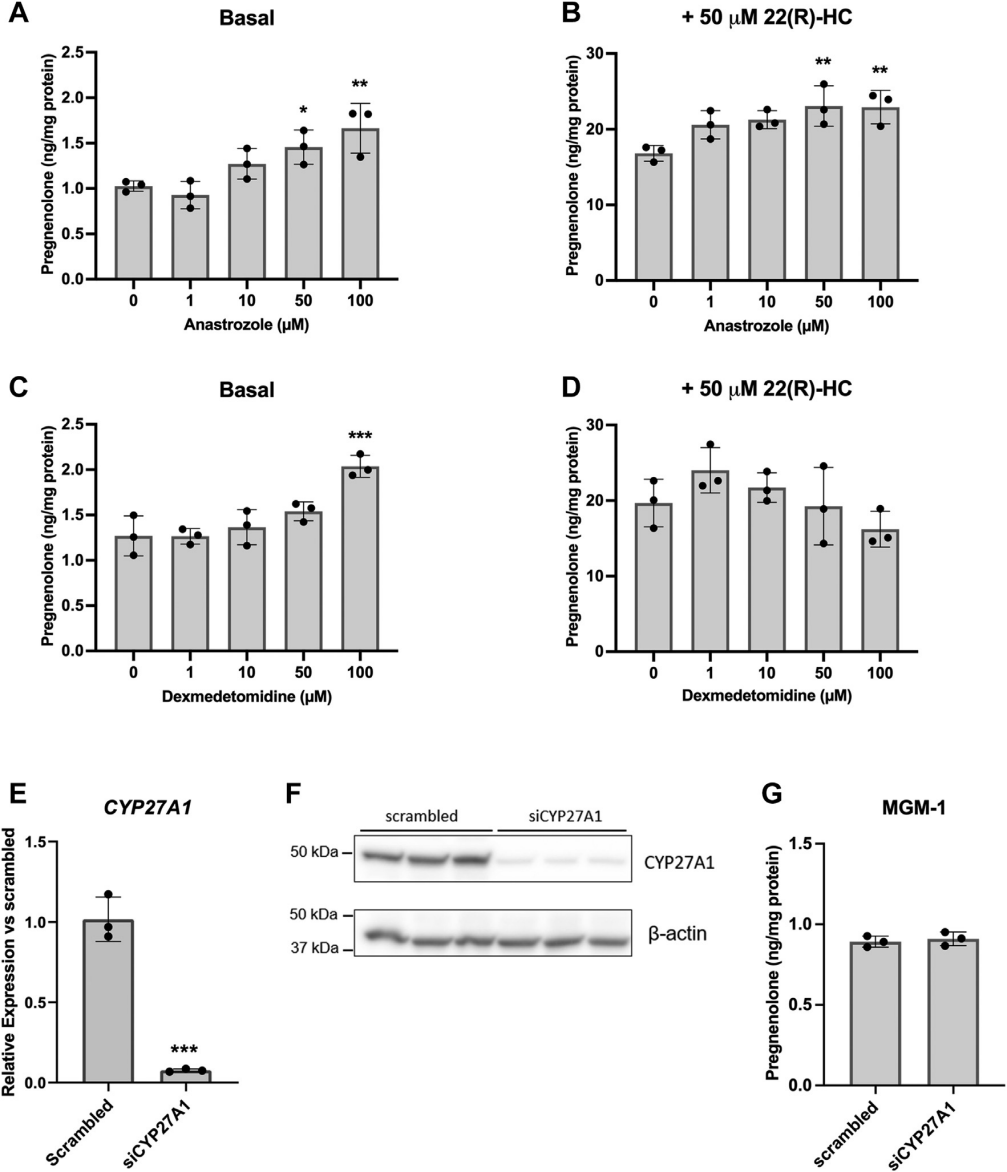
**Effect of CYP27A1 inhibitors and siRNA knockdown on pregnenolone synthesis in MGM-1 cells.***A*–*D*, ELISA measurements of pregnenolone in culture media of MGM-1 cells treated with four different doses of anastrozole (*A* and *B*) or dexmedetomidine (*C* and *D*) for 2 h, under basal conditions (*A* and *C*) or with 50 μM 22(R)-hydroxycholesterol treatment (*B* and *D*). *E*–*G*, siRNA knockdown of CYP27A1 in MGM-1 cells. Knockdown efficiency was determined by qRT-PCR (*E*) and immunoblot (*F*). *G*, ELISA measurements of secreted pregnenolone in transfected MGM-1 cells. Each data point represents the average from one experiment, where each treatment was performed in triplicate within each experiment. Data are presented as mean ± SD, N = 3. Statistics performed compared to no-inhibitor treatment group (*A*–*D*) or scrambled negative transfection control (*E* and *G*). (∗ *p* < 0.05, ∗∗ *p* < 0.01, ∗∗∗ *p* < 0.001). qRT-PCR, quantitative reverse transcription PCR.

We first examined whether CYP27A1 is involved in pregnenolone synthesis, since it is known to metabolize cholesterol (33) and is the most highly expressed mitochondrial CYP450 in MGM-1 cells. We chose two structurally different CYP27A1 inhibitors, anastrozole and dexmedetomidine, both of which were among the top eight tested drugs to inhibit CYP27A1 activity by more than 40% in a study by Mast *et al*. (34). When we treated MGM-1 cells with these CYP27A1 inhibitors, we did not observe inhibitory effects on pregnenolone synthesis, either under basal conditions or treatment with 22(R)-HC (Fig. 1, *A*–*D*). In fact, the higher concentrations of anastrozole and the highest concentration of dexmedetomidine increased pregnenolone production under basal conditions instead. To confirm whether the lack of inhibition may be due to off-target effects of the drugs, we performed siRNA knockdown of CYP27A1 (Fig. 1, *E*–*G*). We observed over 85% knockdown of CYP27A1 expression (Figs. 1, *E* and *F* and S1); however, there was no significant change in pregnenolone production (Fig. 1*G*). Altogether, our data suggest that CYP27A1 is not involved in pregnenolone synthesis in MGM-1 cells.

### Inhibition of CYP1B1 leads to decreased pregnenolone synthesis in glial cells but not in adrenal cells

**Figure 2:**
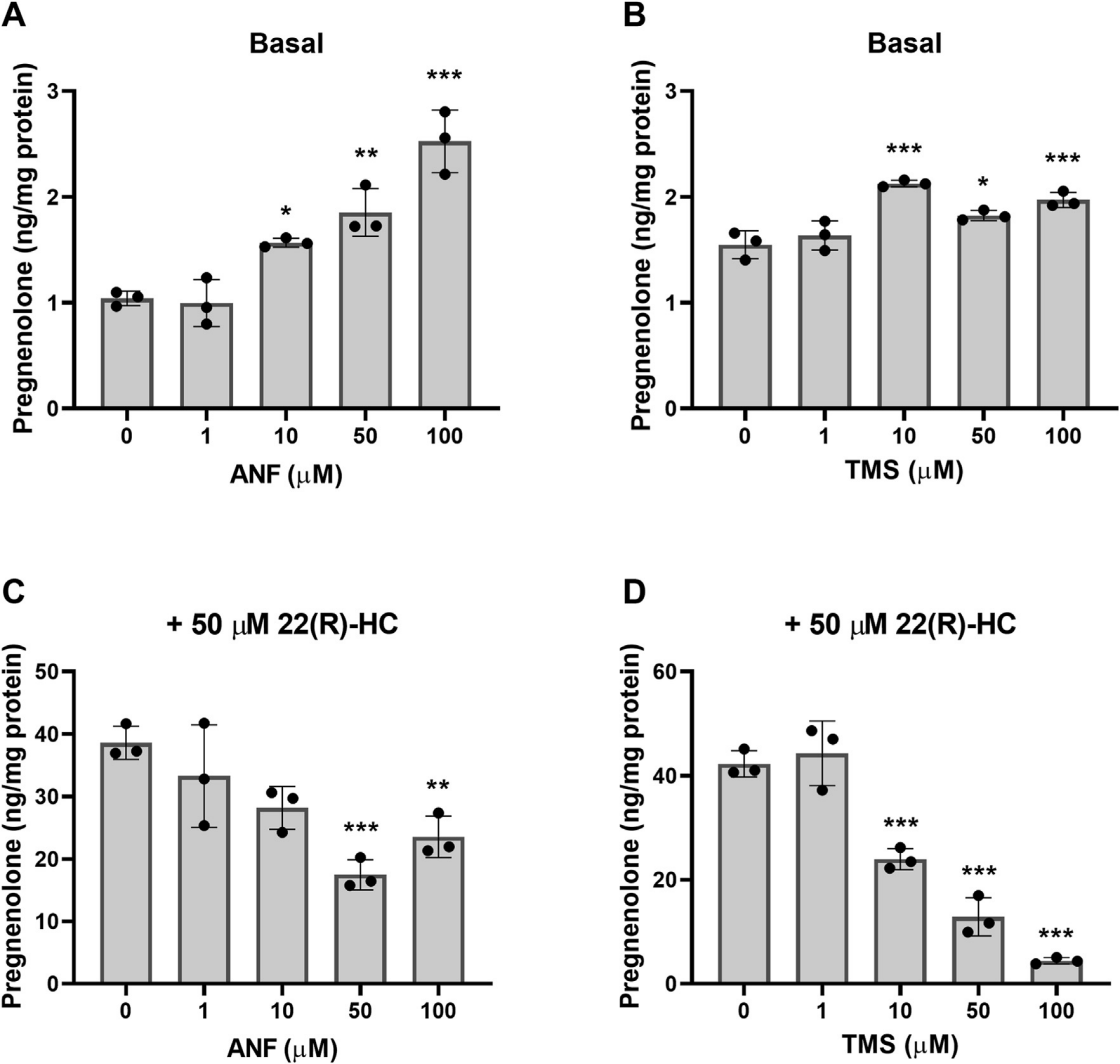
**Effect of CYP1A1/CYP1B1 inhibitors on pregnenolone synthesis in MGM-1 cells.** ELISA measurements of pregnenolone in culture media of MGM-1 cells treated with four different doses of α-napthoflavone (ANF) (*A* and *C*) or 2,4,3′,5′-tetramethoxystilbene (TMS) (*B* and *D*) for 2 h, under basal conditions (*A* and *B*) or with 50 μM 22(R)-hydroxycholesterol (22(R)-HC) treatment (*C* and *D*). Each data point represents the average from one experiment, where each treatment was performed in triplicate within each experiment. Data are presented as mean ± SD, N = 3. Statistics performed compared to no-inhibitor treatment group. (∗*p* < 0.05, ∗∗*p* < 0.01, ∗∗∗*p* < 0.001).

**Figure 3:**
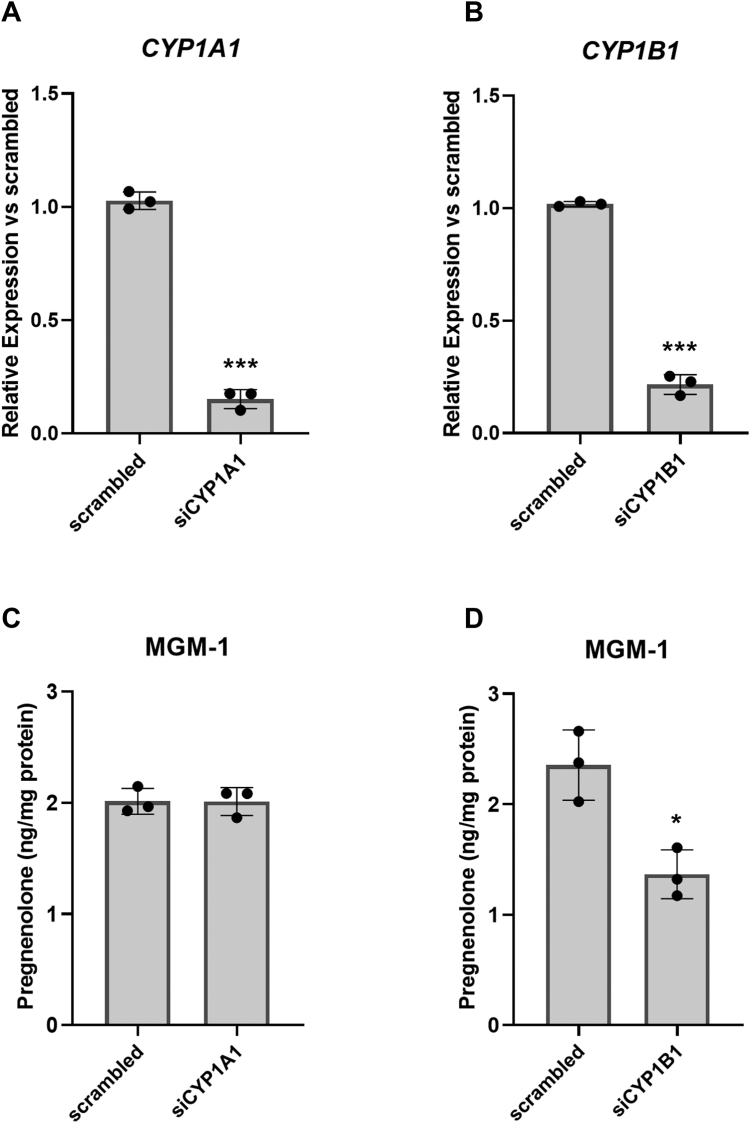
**Effect of CYP1A1 or CYP1B1 siRNA knockdown on pregnenolone synthesis in MGM-1 cells.** Knockdown of *CYP1A1* or *CYP1B1* expression determined by qRT-PCR (*A* and *B*). Pregnenolone synthesis of transfected MGM-1 cells measured by ELISA (*C* and *D*). Each data point represents the average from one experiment, where each treatment was performed in triplicate within each experiment. Data are presented as mean ± SD, N = 3. Statistics performed compared to scrambled negative control. (∗*p* < 0.05, ∗∗∗*p* < 0.001). qRT-PCR, quantitative reverse transcription PCR.

Next, we explored whether CYP1A1 and CYP1B1 are involved in pregnenolone synthesis, as CYP1B1 is the second highest expressed mitochondrial CYP450 in MGM-1 cells. We initially examined the two CYP450s together because most CYP1B1 inhibitors also inhibit CYP1A1 (35). Furthermore, although CYP1B1 is more highly expressed in MGM-1 cells than CYP1A1 (Table 1), our previous findings where ketoconazole increased pregnenolone production in MGM-1 cells suggested the potential involvement of CYP1A1, as ketoconazole has been shown to induce CYP1A1 activity (36). We treated MGM-1 cells with two structurally different CYP1A1/CYP1B1 inhibitors, α-napthoflavone (ANF) and 2,4,3′,5′-tetramethoxystilbene (TMS) (Fig. 2). Although ANF and TMS increased pregnenolone levels under basal conditions (Fig. 2, *A* and *B*), both drugs significantly inhibited pregnenolone production when cells were also treated with 22(R)-HC (Fig. 2, *C* and *D*). To differentiate between the effects of CYP1A1 and CYP1B1 inhibition, we performed siRNA knockdown of CYP1A1 or CYP1B1 in MGM-1 cells. *CYP1A1* and *CYP1B1* mRNA levels were reduced over 80% (Fig. 3, *A* and *B*), but we were unable to determine the precise protein knockdown efficiency due to lack of sensitivity and specificity of commercially available antibodies for CYP1A1 and CYP1B1 (Figs. S2 and S3). Despite a significant reduction in *CYP1A1* expression, pregnenolone synthesis was not altered in *CYP1A1* knockdown cells (Fig. 3, *A* and *C*). On the other hand, knockdown of *CYP1B1* expression significantly reduced pregnenolone production by MGM-1 cells (Fig. 3, *B* and *D*). These findings suggest that CYP1B1 but not CYP1A1 may be involved in pregnenolone synthesis in MGM-1 cells.

**Figure 4:**
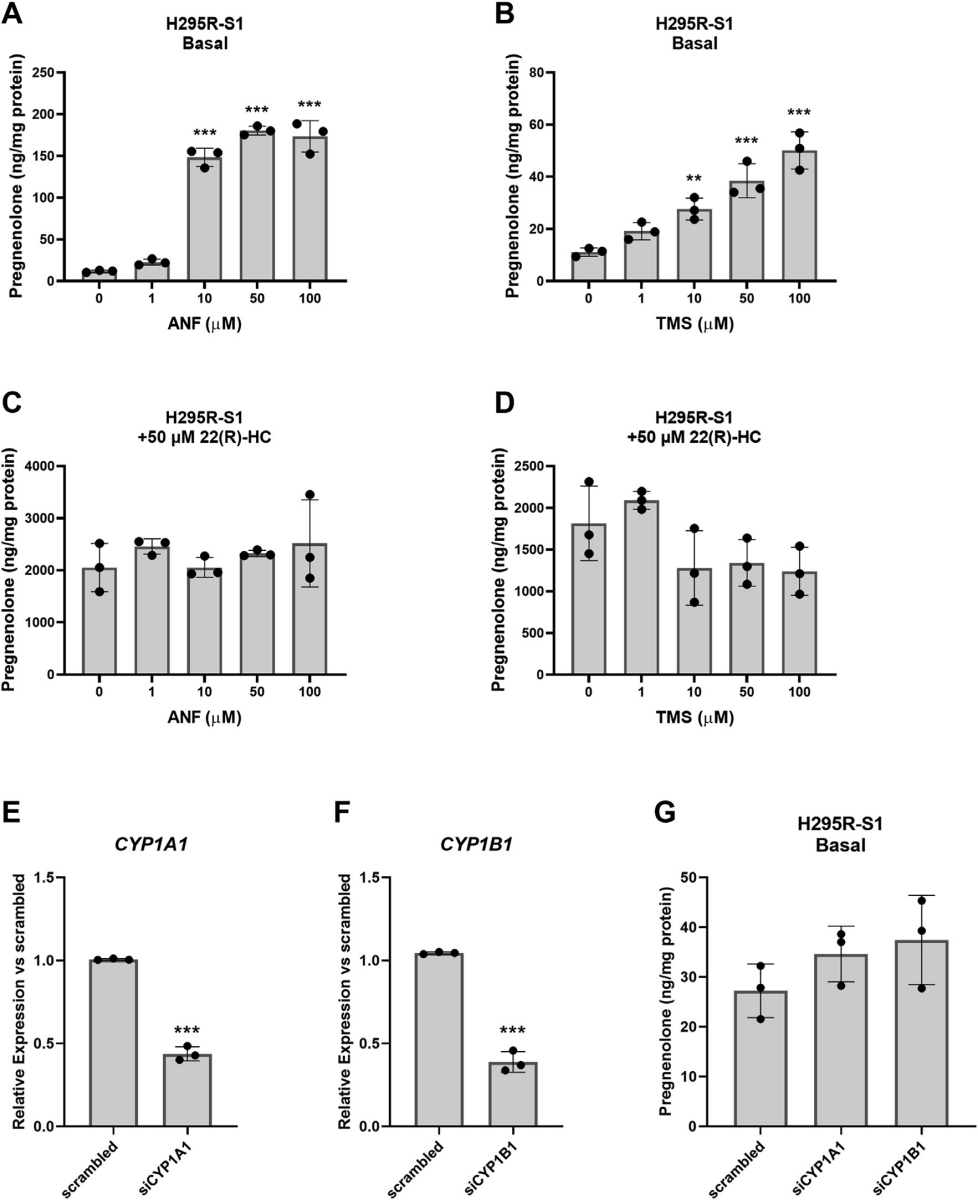
**Effect of CYP1A1/CYP1B1 inhibitors or siRNA knockdown on pregnenolone synthesis in H295R-S1 cells**. *A*–*D*, ELISA measurements of pregnenolone in culture media of H295R-S1 cells treated with different doses of ANF (*A* and *C*) or TMS (*B* and *D*) for 2 h, at basal (*A* and *B*) levels or with 50 μM 22(R)-HC treatment (*C* and *D*). *E*–*G*, knockdown of *CYP1A1* or *CYP1B1 via* siRNA and its effect on pregnenolone synthesis (*G*) in H295R-S1 cells. Knockdown efficiency was determined by qRT-PCR (*E* and *F*). (∗∗*p* < 0.01, ∗∗∗*p* < 0.001). ANF, α-napthoflavone; qRT-PCR, quantitative reverse transcription PCR; TMS, 2,4,3′,5′-tetramethoxystilbene.

To investigate whether CYP1B1 may also be a minor pregnenolone synthesis pathway in peripheral steroidogenic cells, we examined the effects of CYP1A1/CYP1B1 inhibitors and siRNA knockdown on human adrenal cortical carcinoma cells H295R-S1. Similar to MGM-1 cells under basal conditions, the CYP1A1/CYP1B1 inhibitors ANF and TMS significantly increased pregnenolone production in H295R-S1 cells under the same conditions (Fig. 4, *A* and *B*). However, with treatment of 22(R)-HC, ANF, and TMS did not alter pregnenolone synthesis in H295R-S1 cells (Fig. 4, *C* and *D*). Furthermore, neither knockdown of *CYP1A1* (Fig. 4*E*) nor knockdown of *CYP1B1* (Fig. 4*F*) altered pregnenolone production in H295R-S1 cells (Fig. 4*G*). Taken altogether, it appears that CYP1B1 is a major player in the pregnenolone synthesis pathway in human glial cells but not in peripheral steroidogenic cells.

### Overexpression of mitochondria-targeted CYP1B1 leads to increased pregnenolone synthesis in glial cells

**Figure 5:**
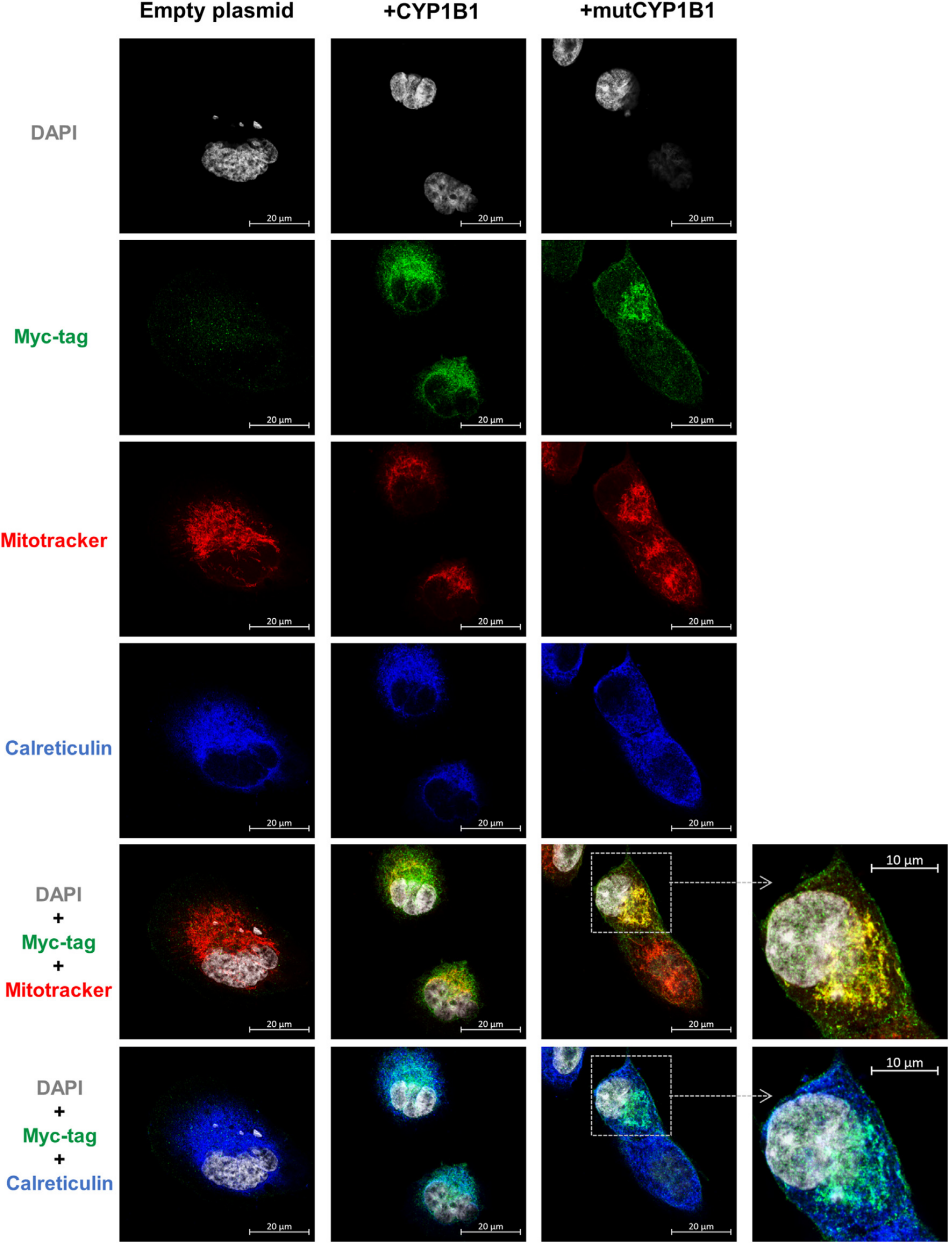
**Localization of WT and mutant CYP1B1 in transfected MGM-1 cells**. Representative confocal images of MGM-1 cells transfected with CYP1B1-Myc or mutCYP1B1-Myc expression vectors. Cells transfected with empty plasmid were used as a negative control. Cells were stained with anti-myc-tag antibody (*green*), mitochondria labeled with MitoTracker Red, endoplasmic reticulum (ER) labeled using anti-calreticulin antibody (*blue*), and nuclei stained with DAPI (*gray*). Images were taken at 63× magnification. Co-localization of overexpressed CYP1B1 with mitochondria appears *yellow*, while co-localization with ER appears *cyan*. DAPI, 4′,6-diamidino-2-phenylindole.

Since inhibition of CYP1B1 led to reduced pregnenolone production by MGM-1 cells, we sought to confirm the involvement of CYP1B1 in pregnenolone synthesis by examining the effects of CYP1B1 overexpression. We transfected MGM-1 cells with a CYP1B1 expression vector (+CYP1B1) with a C-terminal Myc-tag to avoid interfering with the N-terminal localization signals. Although the transfected cells expressed high levels of CYP1B1 protein, we observed that the overexpressed CYP1B1 preferentially localized to the ER (Fig. 5). Since our previous study showed that pregnenolone synthesis occurs in the mitochondria not ER, we also transfected MGM-1 cells with a mutant CYP1B1-Myc expression vector (+mutCYP1B1) where the N-terminus of CYP1B1 was truncated by 34 amino acids to remove the ER-targeting signal and expose the mitochondria-targeting signal. This truncation has been shown to lead to high efficiency of mitochondria import of the enzyme (37). Indeed, we observed that the overexpressed mutant CYP1B1 localized almost exclusively to the mitochondria, with very little ER co-localization (Fig. 5).

**Figure 6:**
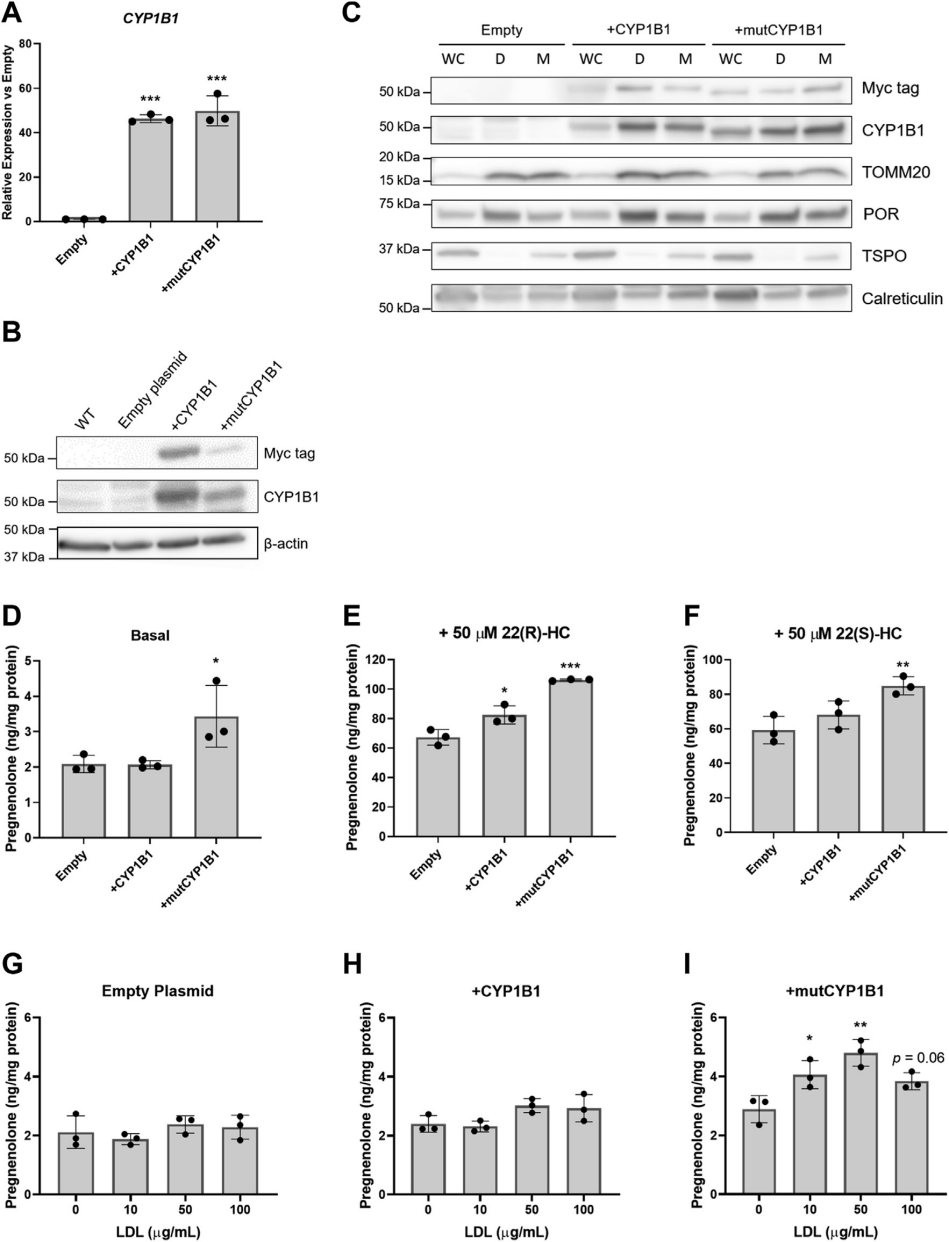
**Pregnenolone production in MGM-1 cells overexpressing WT or mutant CYP1B1**. *A*, qRT-PCR analysis and (*B*) representative immunoblot of CYP1B1 expression in WT MGM-1 cells or MGM-1 cells transfected with empty plasmid, WT CYP1B1 expression vector, or mutant CYP1B1 expression vector. Each lane was loaded with 60 μg of protein lysate. β-actin was used as a house-keeping gene and loading control. *C*, representative immunoblot showing CYP1B1 localization in whole cells (WC), cellular remnants/debris fraction (*D*), or enriched mitochondria fraction (M) in transfected MGM-1 cells. Each lane was loaded with 50 μg of protein lysate. CYP1B1 expression was evaluated using Myc-tag and CYP1B1 antibodies. TSPO (18 kDa; appears as a dimer in human cells) and TOMM20 were used as mitochondrial markers, while POR and calreticulin were used as ER markers. *D*–*F*, ELISA measurements of pregnenolone in culture media of transfected MGM-1 cells under basal conditions (*D*), or with treatment with 50 μM 22(R)-hydroxycholesterol (*E*) or 50 μM 22(S)-hydroxycholesterol (*F*) for 2 h. *G*–*I*, ELISA measurements of pregnenolone in culture media when MGM-1 control (*G*), +CYP1B1 (*H*), and +mutCYP1B1 (*I*) cells were treated with different concentrations of LDL for 24 + 2 h. Data are presented as mean ± SD, N = 3. Statistics performed compared to empty plasmid transfected cells (*A*, *D*–*F*) or 0 μg/ml LDL (*G*–*I*). (∗*p* < 0.05, ∗∗*p* < 0.01, ∗∗∗*p* < 0.001). ER, endoplasmic reticulum; LDL, low-density lipoprotein; qRT-PCR, quantitative reverse transcription PCR.

In MGM-1 +CYP1B1 and +mutCYP1B1 cells, *CYP1B1* mRNA expression was increased more than 40 fold over the negative transfection control (Fig. 6*A*). Although overall CYP1B1 protein expression appeared to be higher in +CYP1B1 cells than in +mutCYP1B1 cells (Figs. 6*B* and S4), CYP1B1 levels were considerably higher in the mitochondria in +mutCYP1B1 cells than in +CYP1B1 cells (Figs. 6*C* and S5). Furthermore, only overexpression of mutant CYP1B1 led to significant increases in pregnenolone synthesis both under basal conditions (Fig. 6*D*) and with treatment of hydroxycholesterols, including the CYP11A1 substrate 22(R)-HC (Fig. 6*E*) and its stereoisomer 22(S)-hydroxycholesterol (22(S)-HC; Fig. 6*F*). Despite the high levels of CYP1B1 expression in +CYP1B1 cells, these cells did not produce more pregnenolone compared to the negative transfection control (Fig. 6, *D*–*F*).

To examine whether CYP1B1 uses cholesterol as the substrate for making pregnenolone, we treated the transfected cells with low-density lipoprotein (LDL), which is used to facilitate cholesterol transport into cells. Unlike adrenal H295R-S1 cells (Fig. S6), LDL treatment did not lead to increased pregnenolone synthesis in MGM-1 control and +CYP1B1 cells (Fig. 6, *G* and *H*). On the other hand, LDL treatment led to a dose-dependent significant increase in pregnenolone production in +mutCYP1B1 cells up to 50 μg/ml (Fig. 6*I*). Taken altogether, our data suggest that mitochondrial CYP1B1 is involved in synthesizing pregnenolone in human glial cells and can use cholesterol as well as hydroxycholesterols as a substrate.

## Discussion

The notion that there may be an alternative pathway for pregnenolone synthesis was proposed 2 decades ago (38, 39), but there have been no studies to provide empirical evidence to show that such pathway or pathways exist or what factors might be involved. Our recent study was the first to show that pregnenolone production in human glial cells is in fact independent of CYP11A1 found in the classical pathway and that another mitochondrial CYP450 must be involved in pregnenolone synthesis instead (22). We continued our investigation here to identify that CYP450. After screening various CYP450s, we identified CYP27A1, CYP1B1, and CYP1A1 as potential enzymes.

Our results quickly ruled out CYP27A1, as neither CYP27A1 inhibitors nor CYP27A1 knockdown *via* siRNA had any inhibitory effects on the pregnenolone synthesis. We then focused on CYP1A1 and CYP1B1, since we found that the inhibitors ANF and TMS significantly inhibited pregnenolone production when human glial cells were co-treated with 22(R)-HC. ANF and TMS have similar IC50s for CYP1B1 (5 nM and 6 nM, respectively), but both drugs also inhibit other enzymes in the CYP1 family (35). In particular, the IC50s for CYP1A1 and CYP1A2 are approximately 300 nM and 3000 nM, respectively, for TMS, while the IC50s for CYP1A1 and CYP1A2 are approximately 6 nM and 60 nM, respectively, for ANF. CYP1A1, CYP1A2, and CYP1B1 can all metabolize steroids downstream of pregnenolone (including estrogens, testosterone, and progesterone (40, 41)), which could explain why we observed increased pregnenolone levels when MGM-1 and H295R-S1 cells were treated with higher concentrations of ANF and TMS under basal conditions. This increase in pregnenolone is likely due to an accumulation caused by inhibition of downstream metabolism rather than stimulation of synthesis. Furthermore, ANF is also known to inhibit CYP19A1 (aromatase) (42), which converts testosterone to estradiol. This could be the reason why the pregnenolone accumulation effects are more apparent in ANF-treated cells compared to TMS-treated cells. With the addition of large amounts of substrate 22(R)-HC, the inhibitory effect of ANF and TMS on synthesis of pregnenolone becomes significant.

Even though ANF and TMS inhibit both CYP1A1 and CYP1B1 activity, we postulated that the decrease in pregnenolone synthesis was more likely due to inhibition of CYP1B1 because *CYP1B1* mRNA expression is at least 10-fold higher than *CYP1A1* in MGM-1 cells. To confirm that the ANF and TMS effects on pregnenolone synthesis were not due to off-target effects, we performed knockdown of CYP1A1 and CYP1B1 in glial cells. Indeed, knockdown of CYP1B1 resulted in decreased pregnenolone production by MGM-1 cells, while knockdown of CYP1A1 had no effect.

To further confirm the involvement of CYP1B1 in pregnenolone synthesis, we overexpressed WT CYP1B1, containing both ER- and mitochondria-targeting signals, as well as a mutant CYP1B1, targeted only to the mitochondria, in glial cells. We found that the overexpressed CYP1B1 was mostly localized to the ER despite having targeting signals for both ER and mitochondria. This suggests that the enzyme responsible for cleaving the ER-targeting signal to expose the mitochondrial signal may not have the capacity to process the signaling peptides with excess *CYP1B1* mRNA (whose levels were increased over 40-fold in +CYP1B1 cells) or that glial cells only direct a small amount of CYP1B1 to the mitochondria under normal conditions. In fact, we observed very little CYP1B1 protein in mitochondria in MGM-1 cells transfected with the empty plasmid. Even though the normal levels of mitochondrial CYP1B1 are low in glial cells, this low level of protein still appears to be functional in producing pregnenolone, as indicated by the detectable levels of pregnenolone in WT MGM-1 cells under basal conditions.

Our data showed that cells with +mutCYP1B1 targeted solely to the mitochondria produced more pregnenolone than +CYP1B1 and control cells, meaning that the mitochondrial environment is necessary for pregnenolone production. This corresponds to our previous finding where knockdown of mitochondria CYP450 co-factor FDXR resulted in reduced pregnenolone synthesis, while knockdown of ER co-factor POR had no significant effect (22). These results are also similar to previous studies showing that CYP11A1 requires the mitochondrial environment to produce pregnenolone, and ER-targeted CYP11A1 could not make pregnenolone from 22(R)-HC (43). We showed that +mutCYP1B1 cells produce increased amounts of pregnenolone when treated with LDL, 22(R)-HC, or 22(S)-HC, indicating that mitochondrial CYP1B1 possesses desmolase activity and can use both cholesterol and hydroxycholesterols as substrates to make pregnenolone. Furthermore, the fact that both 22(R)-HC and 22(S)-HC were converted to pregnenolone suggests that CYP1B1 does not have the same stereospecificity as classical CYP11A1. Our previous study showed that peripheral steroidogenic cells that use CYP11A1 to make pregnenolone do not make increased amounts of pregnenolone when treated with 22(S)-HC (22). Thus, our current results also support our previous speculation that the desmolase activity in glial cells used to make pregnenolone does not appear to be stereospecific.

Although CYP1B1 can metabolize cholesterol and hydroxycholesterols to pregnenolone, its efficiency appears to be much lower than CYP11A1. The MGM-1 +mutCYP1B1 cells produced about 1.5 to 2 times more pregnenolone than control MGM-1 cells, while our previous study showed that MGM-1 +CYP11A1 cells produced almost 40 times more pregnenolone than control cells (22). This is in line with our H295R-S1 results, where inhibition of CYP1B1 did not result in any noticeable decreases in pregnenolone production, unlike MGM-1 cells. Since peripheral steroidogenic cells like H295R-S1 have such high levels of CYP11A1, any pregnenolone made by CYP1B1 would be negligible in comparison.

We speculate that there may be several possibilities for why the brain uses the less-efficient CYP1B1 rather than the classical CYP11A1 for neurosteroid synthesis. First, CYP1B1 has a very broad substrate specificity that includes endogenous substrates such as estrogens, testosterone, progesterone, retinoids, and melatonin, in addition to xenobiotics, including caffeine and theophylline (40, 41, 44, 45, 46, 47, 48). In contrast, CYP11A1 appears to have a much narrower range of substrates, consisting mainly of cholesterol, hydroxycholesterols, 7-dehydrocholesterol, and vitamin D (21). Since the brain does not need to produce large amounts of neurosteroids, which also typically act locally, it may be more efficient to use an already present multifunctional protein rather than expressing a specialized enzyme. This is unlike the periphery where steroids are secreted into the blood to act as hormones and large amounts are needed, thus the efficient CYP11A1 enzyme is more advantageous. Second, the majority of cholesterol in the brain is found in myelin, and additional cholesterol required for brain function is primarily synthesized locally (49). Without access to cholesterol sources from the blood, synthesis of neurosteroids using the less-efficient CYP1B1 may be sufficient and more ideal for cholesterol homeostasis in the brain so as to not deplete local cholesterol sources. This idea is supported by our finding that LDL treatment does not increase pregnenolone production under basal conditions in MGM-1 cells, suggesting that brain cells maintain a low but steady neurosteroid production rate under normal conditions that is unaffected by increased cholesterol availability. Only in MGM-1 +mutCYP1B1 cells where we increased the levels of mitochondrial CYP1B1 substantially above endogenous amounts does the increased cholesterol availability lead to more pregnenolone production. Third, usage of CYP1B1 for neurosteroid synthesis allows for a different regulatory mechanism than CYP11A1. In the periphery, CYP11A1 levels can be stimulated by trophic hormones using cAMP as a messenger (50). However, stimulation of neurosteroid synthesis may not be needed at the same time. Large swings in neuroactive steroid concentration under normal conditions could have detrimental effects on the behavior and brain function. Therefore, having a different stimulation process than the rest of the body for neurosteroid production would allow for a higher degree of regulation and consistency in the brain.

Downstream steroids can also be a feedback mechanism to regulate pregnenolone production, as indicated through the stimulation of pregnenolone synthesis by the presence of DHEA sulfate (51). However, this effect has been attributed to increases in CYP11A1 expression, which is more relevant to the periphery. The regulation of CYP1B1-dependent pregnenolone synthesis by downstream neurosteroids is likely to be more complex compared to CYP11A1 because CYP1B1 is also involved in metabolism of progesterone, testosterone, and estrogens. Furthermore, steroids can regulate the expression of specific microRNAs, one of which (miR-27b) have been shown to alter CYP1B1 expression (52). Therefore, more elaborate studies are needed to elucidate the regulation of CYP1B1-dependent pregnenolone synthesis in the human brain.

Among the cells in the CNS, our study focused on glial cells because they are the brain cells most known to produce neurosteroids. Although the possibility exists that neurons can also synthesize pregnenolone, there is limited evidence in the current literature to support this notion. Very few studies have directly measured pregnenolone biosynthesis activity in isolated neuronal cells, with many studies implying steroidogenic ability of neurons by showing expression of CYP11A1 in rodent neurons (53, 54, 55, 56). In one study where pregnenolone production was explicitly measured, primary rat neurons were found to produce significantly less pregnenolone, given the same amount of substrate compared to oligodendrocytes and astrocytes, but were able to metabolize more downstream neurosteroids (28). This is in line with the fact that adult rodent neurons do not synthesize notable amounts of cholesterol *de novo* and instead rely on glia to supply cholesterol (49), which implies that neurons likely do not have access to sufficient cholesterol to produce significant amounts of pregnenolone and it may be more efficient for glia cells to perform the initial step of steroidogenesis. Nevertheless, the possibility of neurons synthesizing pregnenolone may still be worthy of exploring further, given that CYP1B1 has been shown to be present in neurons in the human brain cortex (57).

Our current study used drug inhibition, siRNA knockdown, and overexpression to assess the involvement of CYP1B1 in pregnenolone synthesis in human glial cells. Although a *CYP1B1* KO model would further strengthen our conclusions, the assessment of neurosteroid production in a *CYP1B1* KO animal model would be difficult. First, CYP1B1 is involved in metabolizing a wide range of endogenous substrates as discussed above, indicating that loss of its function could impact multiple pathways and organ systems. Previous studies have reported that 560 liver genes were differentially expressed in *Cyp1b1* KO mice, which resulted in altered energy homeostasis (58). *Cyp1b1* deletion in mice also led to structural abnormalities in the eye with loss of collagen, resulting in glaucoma (40). Thus, how impaired metabolism of multiple lipids *via CYP1B1* KO would affect the brain is extremely problematic, and the loss of CYP1B1 function could significantly complicate neurosteroid analyses. Second, CYP1B1 KO animals would need to be gonadectomized and adrenalectomized to be able to examine changes in neurosteroids without interference from peripheral steroids, which may be challenging considering CYP1B1 KO animals would already have disruptions in multiple metabolic pathways. To our knowledge, there have not yet been any reports of neurosteroid problems in humans with CYP1B1 mutations. The most common phenotype disease in humans with CYP1B1 mutations is primary congenital glaucoma (59), but the studies in these individuals have not investigated changes in neurosteroids. This is likely due to the fact that peripheral steroidogenic organ activity is intact in these individuals.

In combination with our previous studies (21, 22), our findings may suggest differences in neurosteroidogenesis pathways between human and rodent brains. There have been many reports of Cyp11a1 in the rodent brain but little evidence of CYP11A1 presence in the human brain; meanwhile, there have been more reports of the presence of CYP1B1 in the human brain (57, 60, 61, 62) than the rodent brain (63, 64). It is possible that the human brain mainly uses mitochondrial CYP1B1 to synthesize pregnenolone while the rodent brain uses a combination of Cyp11a1 and Cyp1b1. However, further investigations would be needed to determine whether rodents remain a representative model to study neurosteroidogenesis, as it is unclear whether Cyp1b1 is the major pregnenolone synthesis pathway in rodent brain.

Overall, our study identified an alternative CYP450 to CYP11A1 that can be used by human brain cells to produce the neurosteroid pregnenolone, which provided empirical evidence for the hypothesis that enzymes involved in steroidogenesis in the CNS may be different than those in the classical steroidogenic pathways (39, 65). In combination with our previous work showing that pregnenolone production in the brain is independent of CYP11A1 (22), we found that glial cells use mitochondrial CYP1B1 to synthesize pregnenolone, which changes the approaches that can be taken to modify and treat diseases in the brain that are affected by neurosteroid levels. Furthermore, the involvement of CYP1B1 in an essential step of neurosteroidogenesis has therapeutic implications because CYP1B1 metabolizes a wide range of substrates and its expression is regulated by transcription factors (such as aryl hydrocarbon receptor) that are inducible by xenobiotics (52). This suggests that drugs capable of crossing the blood–brain barrier and are substrates for CYP1B1 could potentially compete with cholesterol or hydroxycholesterols for CYP1B1 activity, which would affect neurosteroid synthesis and likely brain function. Therefore, extra consideration should be taken when CYP1B1 inducers, substrates, or inhibitors are to be used as treatments for their potential effects on neurosteroid synthesis and effects in the brain function.

## Experimental procedures

### Cell culture

The human glioma cell line MGM-1 was a gift from Dr Hiroaki Kataoka (University of Miyazaki). MGM-1 cells were grown in Dulbecco’s modified Eagle medium (Gibco, Thermo Fisher Scientific #11965092) with 10% heat-inactivated fetal bovine serum (Sigma-Aldrich #12306C) plus 100 IU/ml penicillin and 100 μg/ml streptomycin (Gibco, Thermo Fisher Scientific #15140122) at 37 °C and 5% CO_2_. The adrenal cortical carcinoma cell line NCI-H295R (referred to as H295R-S1; #CRL-2128) was purchased from the American Type Culture Collection and grown in Dulbecco’s modified Eagle medium/F-12, GlutaMAX with 2.5% Nu-Serum (Corning, #355100), 100 IU/ml penicillin, 100 μg/ml streptomycin, plus ITS+ Premix Universal Culture Supplement at a concentration recommended by the manufacturer (Corning, #354352). Cells were passaged using trypsin/EDTA 0.25% (Gibco, Thermo Fisher Scientific #25200056), and a maximum of 10 passages were used for all cell lines. For collection of cell pellets for CYP450 screening and antibody testing, MGM-1, MGM-3, NHA, HMC3, MA-10, and Huh7 cells were cultured and pelleted according to our previously published methods (22, 66).

### RNA extraction and qRT-PCR

Total RNA was extracted from cell pellets consisting of >10^5^ cells and DNAse treatment was performed to remove genomic DNA using the RNAqueous-Micro Kit (Thermo Fisher Scientific, #AM1931). RNAs (1000 ng) were reverse transcribed into complementary DNA (cDNA) using PrimeScript RT Master Mix (Takara, #RR036B). qRT-PCR was then performed in 384-well plates using SYBR Select Master Mix (Thermo Fisher Scientific, #4472908) with 100 nM forward and reverse primers (Integrated DNA Technologies). Primers were designed using the NCBI Primer Blast tool, where primer pairs that spanned exon-exon junctions were preferentially selected. Plates were assayed on a CFX384 Touch Real-Time PCR Detection System (Bio-Rad). Sequences of all forward and reverse primers are listed in Table S1 or Dataset S1. For each gene, 20 ng cDNA was used for detection. Data were analyzed using Bio-Rad CFX Maestro software (https://www.bio-rad.com/en-us/product/cfx-maestro-software-for-cfx-real-time-pcr-instruments?ID=OKZP7E15). Relative quantification analysis was performed using the 2^-ΔΔCT^ method, where samples were all compared to negative controls, and β-actin (*ACTB*) was used as a house-keeping gene. The exception is for the CYP450 screening where the 2^-ΔCT^ method was used instead to obtain the relative expression of a particular gene.

### Mitochondria isolation

Crude mitochondria were isolated from cell pellets consisting of >5 × 10^6^ according to previously published methods (67, 68). Briefly, cell pellets were resuspended in 1 ml of a sucrose-based mitochondria extraction buffer (67) and homogenized on ice using a Dounce homogenizer with 12 up-and-down passes of the pestle. The homogenate was then centrifuged at 750*g* for 10 min at 4 °C. The supernatant was collected and the pellet was homogenized and centrifuged again to ensure sufficient disruption of the cells. The supernatant from the two spins were pooled and centrifuged at 10,000*g* for 15 min at 4 °C to obtain a crude mitochondria pellet, which is referred to as the enriched mitochondria fraction. The pellet from the low-speed spin containing other cellular remnants was also collected, which is referred to as cellular debris fraction.

### Cell lysate preparation and Western blotting

Cell pellets consisting of >2 × 10^5^ cells or isolated cell fraction pellets were lysed in radioimmunoprecipitation assay buffer with 2% protease inhibitor (Thermo Fisher Scientific, #A32955). Twenty to 80 μg of total proteins were resolved on 4 to 20% precast polyacrylamide gels (Bio-Rad, #4561096) and transferred to polyvinylidene difluoride membranes (Sigma-Aldrich, #ISEQ00010). After transfer, membranes were blocked for 30 min with blocking solution consisting of 5% bovine serum albumin (Equitech-Bio, #BAH65) dissolved in phosphate buffered saline with Tween 20 (PBST). Membranes were then incubated with specific primary antibodies diluted in blocking solution overnight at 4 °C, washed three times with PBST, incubated with corresponding secondary antibodies for 1 h at room temperature (RT), and washed three times with PBST. Antibodies were detected using Clarity Western ECL Substrate system (Bio-Rad, #1705061) and visualized using a Western blot imaging system (Azure Biosystems, c600). The following primary antibodies were used: anti-CYP27A1 rabbit mAb (Abcam, #ab126785, 1:1000 dilution), anti-CYP1A1 rabbit pAb (Proteintech, #13241-1-AP, 1:1000 dilution), anti-CYP1B1 rabbit pAb (Thermo Fisher Scientific, #PA5-95277, 1:1000 dilution), anti-Myc-tag mouse mAb (Cell Signaling Technology, #20229, 1:1000 dilution), anti-calreticulin rabbit mAb (Abcam, #ab92516, 1:1000 dilution), anti-GAPDH rabbit mAb (Cell Signaling Technology, #2118, 1:2000 dilution), anti-beta actin mouse mAb (Abcam, #ab8226, 1:3000 dilution), anti-TOMM20 mouse mAb (Abcam, #ab56783, 1:1000 dilution), anti-CYP450 reductase (POR) rabbit pAb (Abcam, #ab13513, 1:1000 dilution), and anti-TSPO rabbit pAb (1:1000 dilution, generated according to (66)). The following secondary antibodies were used: WesternSure Goat anti-Rabbit HRP Secondary Antibody (LI-COR, #926–80011, 1:5000 dilution) or WesternSure Goat anti-Mouse HRP Secondary Antibody (LI-COR, #926–80010, 1:5000 dilution). For immunoblots with different cell fractions, total protein staining (Azure Biosystems, #AC2225) was performed according to the manufacturer’s instructions to use as a loading control.

### Cell treatments for steroid measurements

Drugs used to treat cells were purchased from Sigma-Aldrich: 22(R)-hydroxycholesterol (#H9384), anastrozole (#A2736), dexmedetomidine hydrochloride (#SML0956), α-naphthoflavone (#N5757), and TMS (#SMB00388). 22(S)-hydroxycholesterol was purchased from Cayman Chemicals (#21399). Cells were seeded in 96-well plates at a density of 20,000 cells/well. Before treatment, cells were washed three times with PBS to remove steroids from the serum in complete media. Drugs used for treating cells were dissolved in dimethyl sulfoxide or ethanol depending on solubility to make stock solutions with at least 200X concentration. Stock solutions were diluted in serum-free base media to treatment concentrations immediately before treatment. Appropriate solvent controls were made based on the concentration of solvent in the highest concentration treatment condition. Cells were incubated with treatment media for 2 h, after which the media was collected and stored at −80 °C until used for steroid measurement. For LDL treatments, cells were pretreated with LDL from human plasma (Thermo Fisher Scientific, # L3486) for 24 h in complete media prior to the 2 h treatment for steroid measurement. Cells were then lysed with 0.1 M NaOH solution and protein quantity was measured using the Bradford assay (VWR, #97065–020) to normalize steroid measurements. Pregnenolone was measured in media from MGM-1 and H295R-S1 cells using pregnenolone ELISA kits (Abnova, #KA1912).

### Screening of CYP450 enzymes

Microarray data of brain tissues from multiple human donors were obtained from Allen Brain Atlas (https://human.brain-map.org/), searched using the key word “CYP” (27). Information about CYP450 proteins was obtained from UniProt (https://www.uniprot.org/), using the keyword “CYP” (25). Data from both databases were downloaded, cleaned, and analyzed using R to generate a list of CYP450 enzymes that are expressed in the human brain. Primers for these CYP450 enzymes were designed using NCBI Primer Blast and purchased from Integrated DNA Technologies (Table S1 and Dataset S1). qRT-PCR was performed on cDNA from four human glial cell lines (MGM-1, MGM-3, NHA, and HMC3) to evaluate the expression of these CYP450s in glial cells. qRT-PCR results were then merged with CYP450 protein information from UniProt using R. CYP450 enzymes that were expressed in the glial cells and had mitochondrial localization were identified.

### siRNA knockdown of CYP450s

MGM-1 or H295R-S1 cells were seeded into 12-well plates at a density of 75,000 cells/well or into 6-well plates at a density of 150,000 cells/well. The cells were transfected with 5 nM scrambled negative control siRNA duplexes or gene-specific siRNA duplexes (Origene, #SR301124 for CYP27A1, #SR301093 for CYP1A1, and #SR301095 for CYP1B1) using siTran 2.0 transfection reagents (Origene, #TT320001), according to the manufacturer’s instructions. Culture media were replaced 18 h after transfection. Transfected cells in 12-well plates were collected 48 h after transfection for RNA analysis by qRT-PCR. Seventy-two hours posttransfection, transfected cells in 6-well plates were washed and treated with serum-free media for 2 h, after which media was collected for steroid measurement by ELISA and the cell pellet collected for protein quantification and western blotting. Knockdown efficiency was determined by both qRT-PCR and immunoblot analyses.

### CYP450 overexpression

MGM-1 cells were seeded into 12-well plates at a density of 100,000 cells/well. The cells were then transfected with 1 μg of pcDNA3.1(+)-C-Myc mammalian expression vector, WT CYP1B1 cDNA ORF clone in pcDNA3.1(+)-C-Myc vector, or mutant CYP1B1 (mutCYP1B1) cDNA ORF clone in pcDNA3.1(+)-C-Myc vector (GenScript, custom-made plasmids) using Lipofectamine 3000 (Thermo Fisher Scientific, #L3000001) and Opti-MEM (Gibco, Thermo Fisher Scientific, #31985062), according to the manufacturer’s instructions. Sequences for WT CYP1B1 and mutant CYP1B1 ORF clones are listed in Supplementary Information. After 24 h, the culture media were replaced. Transfected cells were then selected with 1 mg/ml geneticin (Gibco, Thermo Fisher Scientific, #10131027) for 14 days qRT-PCR, immunofluorescence, and Western blot were used to confirm CYP1B1 or mutCYP1B1 expression. Steroid measurements for transfected cells were performed as described above.

### Immunocytochemistry

Transfected MGM-1 cells were seeded onto glass coverslips in 24-well plates at density of 10,000 cells/well and allowed to attach overnight. The cells were then incubated with 250 nM MitoTracker Red CMXRos (Thermo Fisher Scientific, #M7512) in complete media for 40 min. Cells were then fixed in 4% paraformaldehyde for 10 min and permeabilized with 0.1% Triton X for 10 min, with three PBS washes between each step. Blocking was then performed for 30 min with 5% donkey serum (Sigma-Aldrich, #D9663) + 0.5% bovine serum albumin (Equitech-Bio, #BAH65) in PBS at RT. Cells were incubated overnight at 4 °C with anti-Myc-tag mouse mAb (Cell Signaling Technology, #20229, 1:500 dilution) and anti-calreticulin rabbit mAb (Abcam, #ab92516, 1:500 dilution) diluted in blocking solution. Following primary antibody incubation, cells were washed three times with PBS and incubated for 1 h at RT with Alexa Fluor 488 goat anti-mouse IgG (H + L) secondary antibody (Thermo Fisher Scientific, #A11001, 1:1000 dilution) and Alexa Fluor 647 goat anti-rabbit IgG (H + L) secondary antibody (Thermo Fisher Scientific, #A21244, 1:1000 dilution). After three PBS washes, coverslips were mounted onto microscope slides with VECTASHIELD Vibrance mounting medium with DAPI (Vector Laboratories, #H-1800). The slides were then imaged at 63× magnification using Zeiss LSM 880 Confocal Microscope with Airyscan Processing.

### Statistics

Statistical analyses of steroid measurements and gene expression changes were performed using GraphPad Prism 9.2.0 (https://www.graphpad.com/). Statistical significance was determined using student’s *t* test or one-way ANOVA followed by Dunnett’s multiple comparison test. For multiple comparison tests, each value was compared to the no-drug solvent control group or transfection negative control.

## Data availability

All data are contained within the manuscript.

## Supporting information

This article contains supporting information.

## Conflict of interest

The authors declare that they have no conflicts of interest with the contents of this article.

## Supporting information

Supplemental data

Screening_Dataset_v1
