# Supplementary material for: Mitochondrial cytochrome P450 1B1 is involved in pregnenolone synthesis in human brain cells
Source: J Biol Chem. 2023 Jul 11;299(8):105035. doi: 10.1016/j.jbc.2023.105035 (PMC10413356; doi:10.1016/j.jbc.2023.105035)
Supplement: Supplemental data [file mmc1.pdf]

Supplementary Information

Mitochondrial cytochrome P450 1B1 is involved in pregnenolone synthesis in human brain cells

Yiqi Christina Lin, Garett Cheung, Zeyu Zhang, Vassilios Papadopoulos*

Department of Pharmacology and Pharmaceutical Sciences, School of Pharmacy, University of Southern California, Los Angeles, CA

Table S1: Sequences of primers used for qRT-PCR.

Gene	Forward Primer	Reverse Primer
CYP1A1	ACAGTGATTGGCAGGTCACG	GTCTCTTGTTGTGCTGTGGGG
CYP1B1	AAGTTCTTGAGGCACTGCGAA	GGCCGGTACGTTCTCCAAAT
CYP27A1	AGGCCAAGTACGGTCCAATG	GTACCAGTGGTGTCCTTCCG
CYP2U1	TCAGCTTCTTTATCGGCCACT	TGCAAACACAACCCCCTTCT
CYP2E1	ACACTACTTGCCTGGAAGCC	CTGCACTGTGCTTTTCCTTCTC
CYP11A1	GCTTTGCCTTTGAGTCCATCA	CTCGGGGTTCACTACTTCCTC
ACTB	CCTTGCACATGCCGGAG	GCACAGAGCCTCGCCTT

Wild-type CYP1B1 Construct.
Genscript Clone ID: OHu20054C
Accession No.: NM_000104.3(ORF Sequence), 1647 bp
Vector name: pcDNA3.1(+)-C-Myc
5' additional: CTCGAGGCCACC, 3' additional: GGGCCC, Start codon: ATG
ORF Sequence:
CTCGAGGCCACCATGGGCACCAGCCTCAGCCCGAACGACCCTTGGCCGCTAAACCC
GCTGTCCATCCAGCAGACCACGCTCCTGCTACTCCTGTCGGTGCTGGCCACTGTGCA
TGTGGGCCAGCGGCTGCTGAGGCAACGGAGGCGGCAGCTCCGGTCCGCGCCCCCGG
GCCCGTTTGCGTGGCCACTGATCGGAAACGCGGCGGCGGTGGGCCAGGCGGCTCAC
CTCTCGTTCGCTCGCCTGGCGCGGCGCTACGGCGACGTTTTCCAGATCCGCCTGGGC
AGCTGCCCCATAGTGGTGCTGAATGGCGAGCGCGCCATCCACCAGGCCCTGGTGCA
GCAGGGCTCGGCCTTCGCCGACCGGCCGGCCTTCGCCTCCTTCCGTGTGGTGTCCGG
CGGCCGCAGCATGGCTTTCGGCCACTACTCGGAGCACTGGAAGGTGCAGCGGCGCG
CAGCCCACAGCATGATGCGCAACTTCTTCACGCGCCAGCCGCGCAGCCGCCAAGTC
CTCGAGGGCCACGTGCTGAGCGAGGCGCGCGAGCTGGTGGCGCTGCTGGTGCGCGG
CAGCGCGGACGGCGCCTTCCTCGACCCGAGGCCGCTGACCGTCGTGGCCGTGGCCA
ACGTCATGAGTGCCGTGTGTTTCGGCTGCCGCTACAGCCACGACGACCCCGAGTTCC
GTGAGCTGCTCAGCCACAACGAAGAGTTCGGGCGCACGGTGGGCGCGGGCAGCCTG
GTGGACGTGATGCCCTGGCTGCAGTACTTCCCCAACCCGGTGCGCACCGTTTTCCGC
GAATTCGAGCAGCTCAACCGCAACTTCAGCAACTTCATCCTGGACAAGTTCTTGAGG
CACTGCGAAAGCCTTCGGCCCGGGGCCGCCCCCCGCGACATGATGGACGCCTTTATC
CTCTCTGCGGAAAAGAAGGCGGCCGGGGACTCGCACGGTGGTGGCGCGCGGCTGGA
TTTGGAGAACGTACCGGCCACTATCACTGACATCTTCGGCGCCAGCCAGGACACCCT
GTCCACCGCGCTGCAGTGGCTGCTCCTCCTCTTCACCAGGTATCCTGATGTGCAGAC
TCGAGTGCAGGCAGAATTGGATCAGGTCGTGGGGAGGGACCGTCTGCCTTGTATGG
GTGACCAGCCCAACCTGCCCTATGTCCTGGCCTTCCTTTATGAAGCCATGCGCTTCTC
CAGCTTTGTGCCTGTCACTATTCCTCATGCCACCACTGCCAACACCTCTGTCTTGGGC
TACCACATTCCCAAGGACACTGTGGTTTTTGTCAACCAGTGGTCTGTGAATCATGAC
CCACTGAAGTGGCCTAACCCGGAGAACTTTGATCCAGCTCGATTCTTGGACAAGGAT
GGCCTCATCAACAAGGACCTGACCAGCAGAGTGATGATTTTTTCAGTGGGCAAAAG
GCGGTGCATTGGCGAAGAACTTTCTAAGATGCAGCTTTTTCTCTTCATCTCCATCCTG
GCTCACCAGTGCGATTTCAGGGCCAACCCAAATGAGCCTGCGAAAATGAATTTCAGT
TATGGTCTAACCATTAAACCCAAGTCATTTAAAGTCAATGTCACTCTCAGAGAGTCC
ATGGAGCTCCTTGATAGTGCTGTCCAAAATTTACAAGCCAAGGAAACTTGCCAAGG
GCCC

Mutant CYP1B1 Construct.
Length: 1554 bp
Vector Name: pcDNA3.1(+)-C-Myc
5' additional: CTCGAGGCCACC, 3' additional: GGGCCC, Start codon: ATG
ORF sequence:
CTCGAGGCCACCATGGTGCATGTGGGCCAGCGGCTGCTGAGGCAACGGAGGCGGCA
GCTCCGGTCCGCGCCCCCGGGCCCGTTTGCGTGGCCACTGATCGGAAACGCGGCGG
CGGTGGGCCAGGCGGCTCACCTCTCGTTCGCTCGCCTGGCGCGGCGCTACGGCGACG
TTTTCCAGATCCGCCTGGGCAGCTGCCCCATAGTGGTGCTGAATGGCGAGCGCGCCA
TCCACCAGGCCCTGGTGCAGCAGGGCTCGGCCTTCGCCGACCGGCCGGCCTTCGCCT
CCTTCCGTGTGGTGTCCGGCGGCCGCAGCATGGCTTTCGGCCACTACTCGGAGCACT
GGAAGGTGCAGCGGCGCGCAGCCCACAGCATGATGCGCAACTTCTTCACGCGCCAG
CCGCGCAGCCGCCAAGTCCTCGAGGGCCACGTGCTGAGCGAGGCGCGCGAGCTGGT
GGCGCTGCTGGTGCGCGGCAGCGCGGACGGCGCCTTCCTCGACCCGAGGCCGCTGA
CCGTCGTGGCCGTGGCCAACGTCATGAGTGCCGTGTGTTTCGGCTGCCGCTACAGCC
ACGACGACCCCGAGTTCCGTGAGCTGCTCAGCCACAACGAAGAGTTCGGGCGCACG
GTGGGCGCGGGCAGCCTGGTGGACGTGATGCCCTGGCTGCAGTACTTCCCCAACCC
GGTGCGCACCGTTTTCCGCGAATTCGAGCAGCTCAACCGCAACTTCAGCAACTTCAT
CCTGGACAAGTTCTTGAGGCACTGCGAAAGCCTTCGGCCCGGGGCCGCCCCCCGCG
ACATGATGGACGCCTTTATCCTCTCTGCGGAAAAGAAGGCGGCCGGGGACTCGCAC
GGTGGTGGCGCGCGGCTGGATTTGGAGAACGTACCGGCCACTATCACTGACATCTTC
GGCGCCAGCCAGGACACCCTGTCCACCGCGCTGCAGTGGCTGCTCCTCCTCTTCACC
AGGTATCCTGATGTGCAGACTCGAGTGCAGGCAGAATTGGATCAGGTCGTGGGGAG
GGACCGTCTGCCTTGTATGGGTGACCAGCCCAACCTGCCCTATGTCCTGGCCTTCCTT
TATGAAGCCATGCGCTTCTCCAGCTTTGTGCCTGTCACTATTCCTCATGCCACCACTG
CCAACACCTCTGTCTTGGGCTACCACATTCCCAAGGACACTGTGGTTTTTGTCAACC
AGTGGTCTGTGAATCATGACCCACTGAAGTGGCCTAACCCGGAGAACTTTGATCCAG
CTCGATTCTTGGACAAGGATGGCCTCATCAACAAGGACCTGACCAGCAGAGTGATG
ATTTTTTCAGTGGGCAAAAGGCGGTGCATTGGCGAAGAACTTTCTAAGATGCAGCTT
TTTCTCTTCATCTCCATCCTGGCTCACCAGTGCGATTTCAGGGCCAACCCAAATGAG
CCTGCGAAAATGAATTTCAGTTATGGTCTAACCATTAAACCCAAGTCATTTAAAGTC
AATGTCACTCTCAGAGAGTCCATGGAGCTCCTTGATAGTGCTGTCCAAAATTTACAA
GCCAAGGAAACTTGCCAAGGGCCC

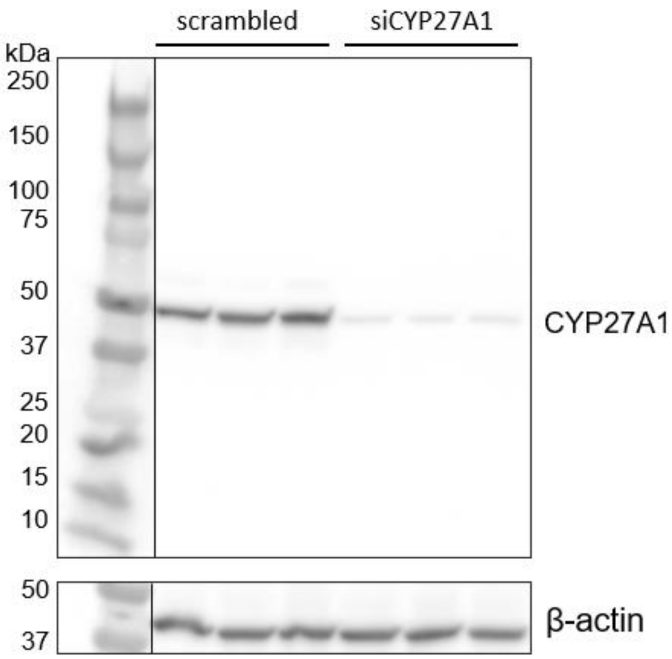

Figure S1: Full membrane image of Figure 1F.

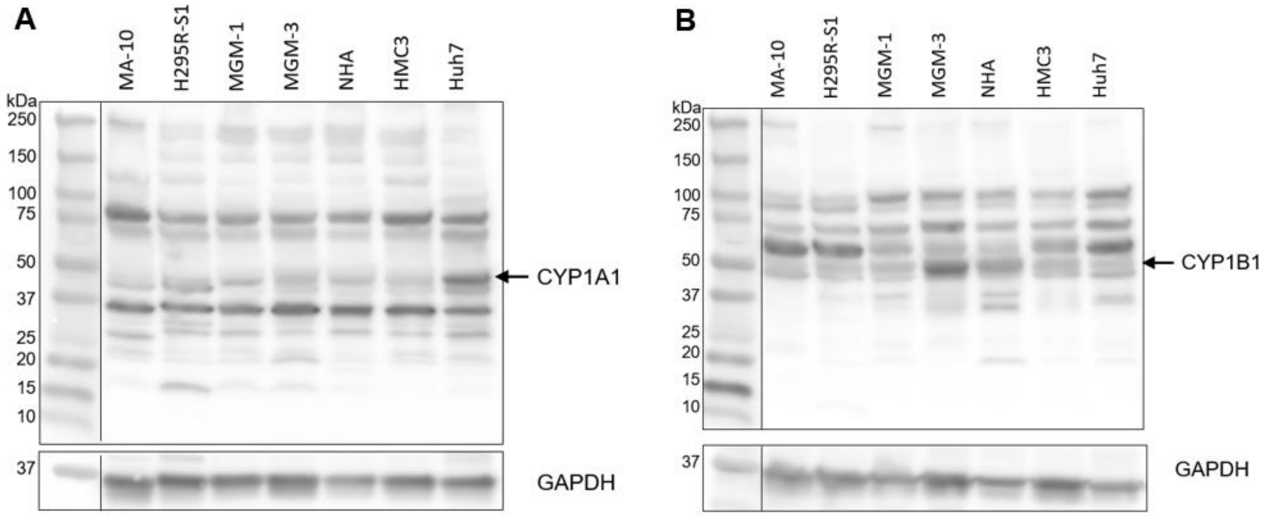

Figure S2: Validation of CYP1A1 and CYP1B1 antibodies for immunoblot. Eighty (80) µg of total protein were loaded into each lane. Both CYP1A1 and CYP1B1 appear as 50 kDa bands on immunoblots. (A) Huh7 human hepatocellular carcinoma cell line was used as positive control for CYP1A1. (B) For CYP1B1, intensity of band at 50 kDa correlates to the RNA expression of CYP1B1 in the glial cell lines (Table 1). CYP1B1 antibody was further validated using MGM-1 +CYP1B1 cells (Figure 6B, Figure S4).

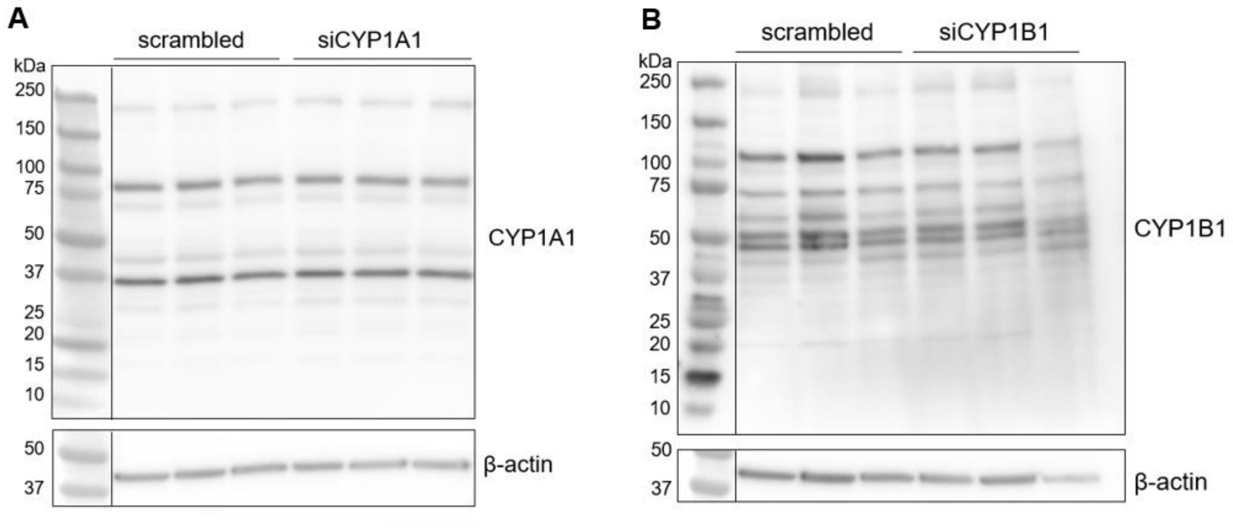

Figure S3: Immunoblots of CYP1A1 and CYP1B1 knockdown in MGM-1 cells. Forty (40) µg of total protein were loaded into each lane. Endogenous expression of CYP1A1 and CYP1B1 was too low for the antibodies to detect knockdown of protein expression, given that antibodies resulted in many non-specific bands.

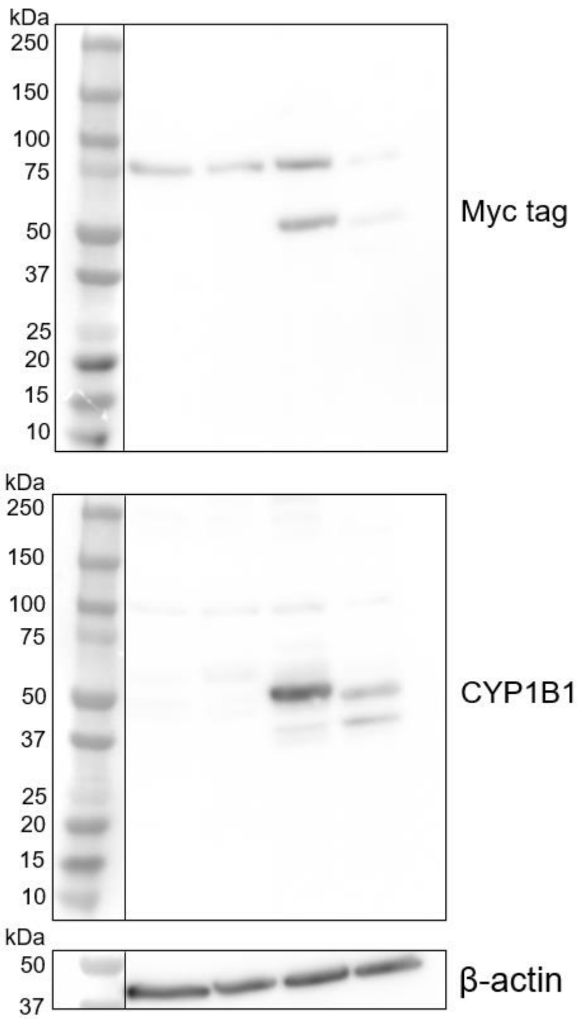

Figure S4: Full membrane images of Figure 6B.

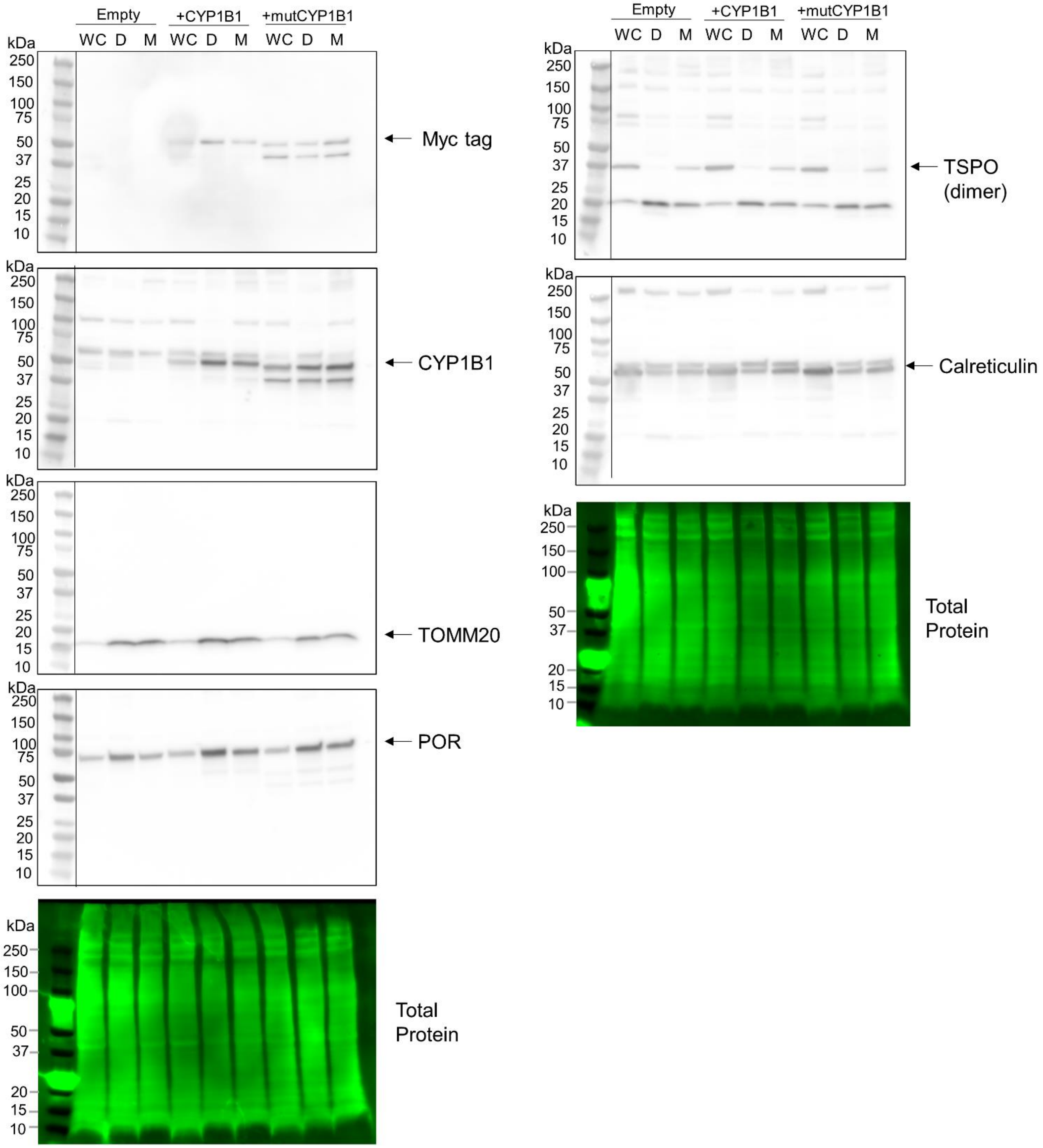

Figure S5: Full membrane images and total protein normalization for immunoblots in Figure 6C. The same cell fraction extracts were diluted to make samples containing 50 µg total protein, which were loaded into two separate gels for western blotting. Identical samples were assessed using two separate immunoblots to prevent over-stripping of the membranes, which can lead to high backgrounds.

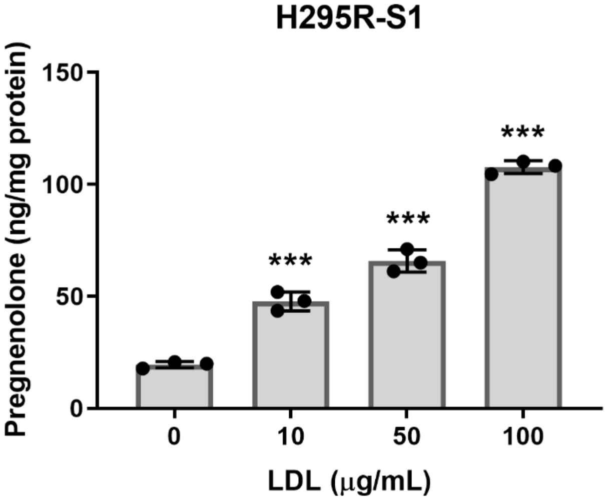

Figure S6: Pregnenolone production in LDL-treated H295R-S1 cells. ELISA measurements of pregnenolone in culture media when H295R-S1 cells were treated with different concentrations of LDL for 24 + 2 h. Data are presented as mean ± SD, N=3. Statistics performed compared to 0 μg/mL LDL. (*** $p < 0.001$)
